# COVID-19 Pandemic: Public Health Risk Assessment and Risk Mitigation Strategies

**DOI:** 10.3390/jpm11121243

**Published:** 2021-11-23

**Authors:** Dae-Young Kim, Surendra Krushna Shinde, Saifullah Lone, Ramasubba Reddy Palem, Gajanan Sampatrao Ghodake

**Affiliations:** 1 Department of Biological and Environmental Science, Dongguk University-Seoul, 32 Dongguk-ro, Ilsandong-gu, Goyang-si 10326, Gyeonggi-do, Korea; sbpkim@dongguk.edu (D.-Y.K.); shindesurendra9@gmail.com (S.K.S.); 2 Interdisciplinary Division for Renewable Energy and Advanced Materials (iDREAM), National Institute of Technology (NIT), Srinagar 190006, India; saifullah.lone@gmail.com; 3 Department of Medical Biotechnology, Dongguk University-Seoul, 32 Dongguk-ro, Ilsandong-gu, Goyang-si 10326, Gyeonggi-do, Korea; palemsubbareddy@gmail.com

**Keywords:** pandemic crisis, SARS-CoV-2, coronavirus, risk assessment, risk mitigation, administrative controls, engineering controls

## Abstract

A newly emerged respiratory viral disease called severe acute respiratory syndrome coronavirus-2 (SARS-CoV-2) is also known as pandemic coronavirus disease (COVID-19). This pandemic has resulted an unprecedented global health crisis and devastating impact on several sectors of human lives and economies. Fortunately, the average case fatality ratio for SARS-CoV-2 is below 2%, much lower than that estimated for MERS (34%) and SARS (11%). However, COVID-19 has a much higher transmissibility rate, as evident from the constant increase in the count of infections worldwide. This article explores the reasons behind how COVID-19 was able to cause a global pandemic crisis. The current outbreak scenario and causes of rapid global spread are examined using recent developments in the literature, epidemiological features relevant to public health awareness, and critical perspective of risk assessment and mitigation strategies. Effective pandemic risk mitigation measures have been established and amended against COVID-19 diseases, but there is still much scope for upgrading execution and coordination among authorities in terms of organizational leadership’s commitment and diverse range of safety measures, including administrative control measures, engineering control measures, and personal protective equipment (PPE). The significance of containment interventions against the COVID-19 pandemic is now well established; however, there is a need for its effective execution across the globe, and for the improvement of the performance of risk mitigation practices and suppression of future pandemic crises.

## 1. Introduction

Coronaviruses were generally not considered highly infectious to humans before 2002; however, after the occurrence of the severe acute respiratory syndrome (i.e., SARS; 2002) [1,2], Middle East respiratory syndrome (i.e., MERS; 2015) [3,4] and COVID-19 pandemic attracted serious attention of the scientific community and public health authorities. SARS-CoV-2 is the ninth known coronavirus to cause infections in humans, causes severe respiratory illness, and breathing discomfort. Its symptoms are similar to those of pneumonia and seasonal Influenza virus, as well as some other coronaviruses [5,6]. Notably, the worldwide spread of this virus was observed within a few months of its first appearance in December 2019 at Wuhan, China [7]. The SARS-CoV-2 outbreak has created a prolonged global public health and economic crisis and created confusion over the need for travel bans and border closures, the closure of educational institutions and businesses, and the implementation of preventive measures [8].

The SARS-CoV-2 virus quickly disseminated worldwide and caused unprecedented public health emergencies. The World Health Organization (WHO) announced COVID-19 as a global pandemic on 11 March 2020. The symptoms of this virus vary widely among individuals, with some patients being completely asymptomatic and others developing severe symptoms together with fever, cough, and fatigue [9]. Many experts agree that the total number of confirmed cases worldwide so far has mainly been underestimated because of the occurrence of asymptomatic patients; further, patients with mild symptoms recover naturally and remain undetected or unaccounted for [10]. The SARS-CoV-2 disease risk is also associated with epidemiological factors, host status (immunity, heredity, age, and overall health), exposure to contaminated surfaces, host tropism, host cell receptors, pathogenesis, etc. [11,12]. Such factors make it even more challenging to quantify the global estimate of existing infections and recoveries. Our previous report described the significance of the biological characteristics of SARS-CoV-2 and its biomarkers to develop diagnostics, point-of-care (POC) testing and surveillance measures [13].

This article covers the significance of risk assessment, which accounts for multiple epidemiological factors associated with the SARS-CoV-2 pandemic (e.g., host range, viral dose, surface survival rate, and some others). The COVID-19 virus is predominantly an airborne disease [14]—the risk of transmission is greatly dependent on the distance of an infectious source [15], ventilation [16], and quality of the face mask; besides, the concentration of the virus in respiratory droplets defines the viral load and a hidden risk of infection from asymptomatic patients [17]. Risk assessment perspectives are thus essential to ensure the safety of viral research laboratories, improve public awareness, as well as ensure the safe disposal of biohazard materials and the overall safety of healthcare workers and officials [18,19]. The risk assessment and mitigation perspectives are intended to design safety measure strategies and potential preventive measures and policymaking decisions.

During the COVID-19 pandemic, personal- and community-level mitigation practices have played a pivotal role in slowing down viral transmission rates and ensuring stability for public healthcare systems [20]. Herein, we analyze prospective data accessible in the literature and use it to describe public health risk factors. We derive key strategies to convey effective mitigation measures to reduce stress on the public healthcare system. Thus, this report has several strengths in terms of public health risk assessment and implementation of mitigation strategies. Lastly, our report emphasizes the efficacy of administrative and engineering controls and enforcing both public interventions, such as social distancing and vaccination and personal interventions, such as face masks. These strategies would reduce the risk of further spread and would be relevant to almost all settings and unsettled contexts, including countries that need additional risk mitigation strategies, such as mass level vaccination, which seems unachievable in the near future.

## 2. Risk Assessment Perspective

### 2.1. Risk Group

The WHO and the National Institutes of Health (NIH) have established frameworks to organize infectious organisms and their toxins into four risk groups based on the risk in humans, adverse impacts on public health, and status of preventative measures and treatment options. In turn, these risk groups are further classified based on human mortality rates, public health risks, and pharmaceutical interventions, preventive measures, and administrative controls [21,22,23,24]. The coronaviruses outbreak SARS (2002–2004), and MERS (occurred in 2015) were classified as Risk Group 3 pathogens according to NIH authorizations [25]. Likewise, the COVID-19 has posed public health and public safety risk, and the European Commission does classify this virus as a Risk Group 3 pathogen [25]. The SARS-CoV-2 risk has been partly reduced by the rapid advancement of diagnostic kits and vaccination; however, delay in drug development is still the basis for the virus’ status as a high-Risk Group pathogen. So far, the COVID-19 pandemic has posed an ominous threat to public health as a result of rapid worldwide spread and impact on human health, in addition to massive economic and social disruption [26].

An integrated risk assessment and inclusive contact tracing must be executed for international and domestic travelers, using air passenger itinerary data, surveillance data, global positioning system (GPS) data, and individual case reports [27]. According to a public report estimate, approximately 60,000 air passengers traveled from Wuhan to more than 382 cities worldwide in the early days of emergence, among which 850 were the carrier of the SARS-CoV-2 virus before lockdown measures were implemented in Wuhan, China. Most of those travel destinations were in Asian countries; however, some other individuals traveled to central Europe, Australia, and the United States, thus supporting strong correlations between the predicted travel risks and the reported cases. Let us suppose that international travel restrictions had been timely implemented, particularly on residents of Wuhan, China, in the early days of COVID-19 emergence. If so, it can be concluded that we could have successfully avoided the virus’ spread elsewhere in the world and perhaps reduced the risk of a pandemic outbreak [28].

### 2.2. Host Range

The host range of any infection can be used to determine whether it infects a particular host or is likely to be diverse. Therefore, it is essential to understand how an animal virus mutates or evolves to cause human–human infections, resulting in a global pandemic. The answers to such questions lie in the biological characteristics of betacoronaviruses, viral genome mutations, and the evolution of new viral progenies that enabled COVID-19 virus infections in animal species, including humans and different host cell types [29].

Coronaviruses and some other RNA viruses are known for their rapid mutation rate [30,31]. This characteristic feature allows betacoronaviruses to enable a rapid diversification rate, thus occasionally rendering either more virulent progenies or generating nonviable variants [32,33]. Evolutionary epidemiology suggests that viral adaptation may occur in a new host range and is driven mainly by stochastic mutations [34], which may encounter the host or miss the target; however, it limits our capacity to anticipate evolutionary changes and the risk of new variants.

The risk of viral diseases is never-ending; thus, it is challenging us by emerging, re-emerging, or resurging unpredictably. Human beings and animals are confronting an intractable challenge from time to time due to infectious viral diseases and public health emergencies. Human infiltration, ecological concerns, habitat loss, and viral-related factors—including natural selection, mutation, and new variants—are the causal factors in the emergence or re-emergence of novel viral diseases [35]. The expansion of the viral host range results in a surplus of natural host reservoirs, which often leads to different variants, and the emergence or resurgence of viral diseases continues [36]. The S protein is a critical target region for the formation of new evolutionary variants. Specifically, this protein recognizes different host species and different host cell target proteins within a specific host and other host cell receptors; this phenomenon is referred to as tropism [37]. The antigenic variations in S proteins for both the SARS-CoV-2 and SARS viruses have been examined to determine their antigenicity [38]. Approximately six epitopes (CVADYSVLY, RISNCVADY, RSFIEDLLF, MTSCCSCLK, VLKGVKLHY, and RVDFCGKGY) match with SARS-CoV-2 and SARS S protein [39]. Variations in the S protein amino acid residues and the distinctive biological features of the SARS-CoV-2 can be a basis for an infection less severe than the MERS infection [40]. However, unlike MERS, the SARS-CoV-2 virus successfully caused a global pandemic crisis because of its higher transmissibility [39].

The spread of viruses to different species must lead to new reservoirs in animals and, after the mutations, it must have eventually enabled them to target human hosts. Given the rapid global spread of the COVID-19, it is likely that this virus must have mutated several times, with a high probability of variant recurrence [41,42]; it has been more challenging to develop vaccines and therapeutics [43]. A recent study reported quantitative data on tropism, cell damage, and replication kinetics in the SARS-CoV-2 virus [44]. Peridomestic animals or wildlife species must have served as stable transitional reservoirs, thus improving the likelihood of the SARS-CoV-2 being accidentally transmitted to human hosts [45,46,47]. Public safety surveillance and epidemiological reports suggested that MERS originated from dromedary camels in Qatar. Domesticated animals act as a stable reservoir for a viral disease, which eventually infects human hosts [48,49].

Moreover, epidemiological investigations suggest that the SARS virus also had an animal origin and is known for rapid transmission, resulting in a sizable pandemic. Furthermore, structural and serological studies have indicated that this virus was initially carried by palm civets (*Paguma larvata*), as confirmed by samples of live animals infected with SARS [50,51]. Therefore, further research is required to identify changes in the biological features in the future SARS-CoV-2 variants and their susceptibility to infect a broad host range and further development in tropism [52,53,54].

The current SARS-CoV-2 outbreak is linked to a wet market in Wuhan, China; therefore, it is widely thought that the wild animals traded might have been a source for the zoonotic transmission of COVID-19 [55]. Such concern also raised the question of whether SARS-CoV-2 spreads from humans to pet animals and, eventually, to wildlife; as a consequence, it will generate new reservoirs besides those that already exist [56,57]. It was found that outbred cats were more susceptible to the SARS-CoV-2 virus, and airborne transmission was reported in cats and ferrets [58]. This report further illustrated that dogs with low susceptibility did not support viral replication of SARS-CoV-2 and that chickens, ducks, and pigs were also not susceptible. This observation suggests that there is the possibility that a few more potential wild animal reservoirs elsewhere in the world exist [58,59]. The Malayan pangolin population is also more susceptible to various coronaviruses; therefore, it is also considered a potential host reservoir for SARS-CoV-2 [60]. Strict measures against the trade of non-farmed animals, high standards of hygiene practices, and a regulatory framework for the wet market would help avoid the emergence of viral diseases and perhaps prevent predictable zoonotic transmissions [60,61,62].

### 2.3. Possible Transmission Routes

SARS, MERS, and SARS-CoV-2 are airborne coronaviruses that mainly spread via coughing, sneezing, and talking by a virus carrier individual. SARS-CoV-2-infected humans expel differently sized aerosolized droplets into the air with a great force during coughing. Tiny-sized aerosol droplets (<4 μm) readily travel relatively long distances and, within reach of nearby individuals, result in airborne viral transmission and being prone to high risk, particularly in indoor settings [63]. On the other hand, large droplets fall in close proximity and contaminate those surfaces; there is the possibility of direct contact or surface transmission; further details can be found in a report on transmissibility and transmission routes [64]. Immunocompetent individuals are also susceptible to higher viral loads [65], become infected simply via touching contaminated surfaces with mucous droplets, have viable SARS-CoV-2 virus, and possibly transmit the virus if coming in contact with the nose, eyes, or mouth (i.e., indirect contact) [66].

Person-to-person aerosol-mediated airborne transmission occurs most frequently in large interacting groups, including family members, friends, neighbors, tourists, shoppers, healthcare/hospital workers, and other settings; proximity favors direct transmission [67]. However, a recent report suggested that SARS-CoV-2 viral particles in aerosol droplets can remain viable in cold air, causal of rapid airborne transmission, especially in the winter season [68]. Aerosol droplets from SARS-CoV-2-infected people may pose a severe threat even at considerably long distances and in enclosed spaces, particularly if they lack proper ventilation [67]. Breathing and loud talking also produce smaller aerosol particles, similar to those reported by Anfinrud et al. [69].

The transmission and spread risks of the SARS-CoV-2 virus can be avoided by isolation of quarantine measures if infected individuals show symptoms. However, some infected individuals stay infectious while asymptomatic, and those patients continue the risk of silent dissemination [70]. Some infected individuals are more susceptible to severe coughing and produce more aerosol particles than others, thus acting as super-spreaders. The diameters of aerosol particles fall within the micron range hardly affected by gravity [71]; such droplets are prone to disperse or travel by airflow [72]. After the emergence of the SARS-CoV-2 pandemic, routes of transmission have been a central topic of debate. So far, inhalable aerosol droplet-mediated airborne transmission is being considered as the primary basis for the manifestation of a global pandemic [73].

Some other possible airborne transmission routes for the SARS-CoV-2 virus may be a rare event via air medium containing solid particulate matter (PM), dust particles, and air pollutants; so far, it is supposed to be involved in coronavirus infection [74]. An inhalation of virus-loaded airborne dust and PM can pass the virus into deeper tracheobronchial and alveolar regions, which may rarely pose a risk of infection [75]. The risk of long-distance travel with sustained viability is of great concern since airborne dust particles can provide a sufficient surface area to adsorb SARS-CoV-2 viruses. Therefore, detailed investigations over possibilities of SARS-CoV-2 virus’ adsorption onto dust surfaces, its viability, and transmission risks need to be investigated further for their role in dissemination.

### 2.4. Fomite-Mediated Transmission

However, we should not overlook alternative transmission routes; otherwise, they may have serious ramifications, particularly in indoor settings [76]. Multiple transmission routes are possible for the SARS-CoV-2 infections, including fomites (objects or materials likely to transmit diseases, such as utensils, clothes, and furniture); however, it depends on temperature, humidity, viral load, and some other factors [77]. Therefore, the relative risk of the SARS-CoV-2 transmission via fomite transmission is negligible owing to low viral load or inactivation of viral particles by environmental factors, temperature, and humidity [78,79,80]. Fortunately, a fomite-mediated transmission is a rare event, but it is challenging to decouple it from other possible transmission routes, particularly in the case of transmission by asymptomatic patients. The SARS-CoV-2 can be transmitted to others if healthy individuals contact the contaminated surfaces and touch the nose, mouth, or eyes. Therefore, proper hand hygiene can be an excellent intervention to avoid the fomite route and reduce the risk of transmission [81].

Microbial risk assessment is applicable to quantify and understand the relative risk of fomite-mediated transmission and evaluate the efficiency of preventive actions to lower the risk of COVID-19 [80]. Fomites have given a relatively low contribution to the rapid spread of the COVID-19; however, guidelines need to be followed to avoid the risk and anxiety of infection [64]. Good hand hygiene, including washing hands with soap and 70% alcohol-based hand sanitizers, could reduce the risk of fomite transmission, mainly in the home, healthcare facilities, and community settings [82]. This report suggests the need to develop quantitative models for identifying high-risk objects and effective sanitization practices to reduce the risk, particularly at indoor settings with high priority (e.g., public buildings, treatment centers, testing facilities). A better understanding of disinfectant efficacy on diverse surfaces and their possible side effects, such as toxicity and negative impact on the environment and human health, will allow us to choose optimal disinfection strategies [83].

### 2.5. Surface Survival

Numerous researchers have investigated the surface survival of COVID-19 on various surfaces, including non-porous and porous objects [84,85,86]. These studies reported suggesting the fewer viability of viruses on porous surfaces. Virus persistence is the ability of a virus to maintain its viability onto solid surfaces or in airborne aerosol particles. Since the membrane of enveloped viruses is made up of lipids and proteins, they are known to be more prone to inactivation and desiccation than those of non-enveloped viruses [87]. Enveloped viruses lose their viability once the envelope disrupts. The aerosol transmission route was also a central driver for the spread of SARS during 2002–2003 [88]. Both SARS-CoV-2 and SARS viruses have similar concerns about viability; long-term survival in the air or on surfaces is the basis for spreading the current SARS-CoV-2 pandemic [87]. Doremalen et al. recently reported that both SARS viruses could remain viable for a few hours on concrete surfaces [68]. Their viability on plastic, steel, cardboard, and copper surfaces is about 15, 13, 8, and 3 h, respectively [25]. However, impermeable non-porous surfaces support the extended viability of the virus, depending on the temperature and relative humidity. The relatively rapid inactivation of SARS-CoV-2 viruses is possible onto the porous surfaces compared to the non-porous; it attributes to the faster evaporation of aerosol droplets and prompt capillary action by porous structures [89].

### 2.6. Wastewater-Based Epidemiology

Recent studies highlighted the significance of wastewater-based epidemiological investigations in performing SARS-CoV-2 prevalence and community surveillance, particularly after the establishment of the pandemic crisis [90]. Thus, wastewater-based epidemiology has raised several questions for handling the SARS-CoV-2 pandemic that needs to be addressed. Virus RNA can be detected in saliva, urine, and stool samples of COVID-19-infected patients; however, it is unclear whether the fecal transmission route is possible. Particularly, trace RNA residues of SARS-CoV-2 were detected in fecal samples for more than 30 days in recovered patients, wherein respiratory test results were negative, indicating the shedding of RNA residues via urine or urine fecal matter after the complete recovery of the patient. However, it would be essential to explore the possibility for the SARS-CoV-2 virus survival in wastewater settings and perform community surveillance practices [91]. There is a need for further efforts to examine the survival of COVID-19 in some other environment settings in addition to the effect of wastewater treatments on the virus’ fate [91].

In addition to this, there is a need to develop robust protocols to make it easier to concentrate and quantify enveloped viruses in water samples, which is pivotal [92]. At present, several research efforts concerning the detection and quantification of enveloped viruses in water samples employ some methods applicable for non-enveloped viruses. The SARS-CoV-2 virus has been detected successfully for trace RNA in various wastewater samples using several concentration protocols [93]. However, wastewater sampling suffers from several limitations, including delays in sampling and testing, viral inactivation during transportation or depending on temperature, dilution caused by precipitation, variability in the sample, and a lack of sophisticated detection systems [94]. Despite these factors limiting wastewater surveillance, it holds tremendous potential as an inexpensive type of widespread monitoring that can detect hotspots before they turn into outbreaks, inform recovery guidance and avoid the emergence or resurgence of the SARS-CoV-2 [95]. Further efforts are required in the field, including policy reforms, ethical practices, sophisticated protocols useful for measuring virus concentrations in wastewater, and accurate estimation of disease prevalence and community surveillance [94,96].

### 2.7. Reproduction Number

The transmissibility of a viral disease is an essential factor in estimating the virus’ ability to disseminate from an infected person to another host or healthy individual. To assess this, the analysis of “R naught” or “R0”, the reproduction number, is mostly used to determine the likelihood of an epidemic crisis or its severity. Therefore, the R0 can be used to explain how any novel or emerging infection could spread in a susceptible population. Therefore, this parameter is a fundamental concept in the studies of the epidemiology of infectious viruses and other contagions, thus highlighting the instrumental role in understanding any contagious disease that has the potential of global spread and causing the pandemic crisis [97]. The given infections are likely to fade quickly at R0 values below 1.0. If the R0 value is about 1.0, the disease will remain in an exposed or low-immunity population. If the R0 value exceeds 1.0, it may cause an epidemic or outbreak such as SARS-CoV-2. According to the WHO and the data collected from an exposed population in Wuhan during the initial episodes of SARS-CoV-2 spread, an average R0 value was approximately 2.3 and reported even higher R0 values [98]. Nevertheless, other independent assessments predicted the R0 for SARS-CoV-2 to range from 1.8 to 3.6, consistent with the WHO estimate [99].

**Figure 1:**
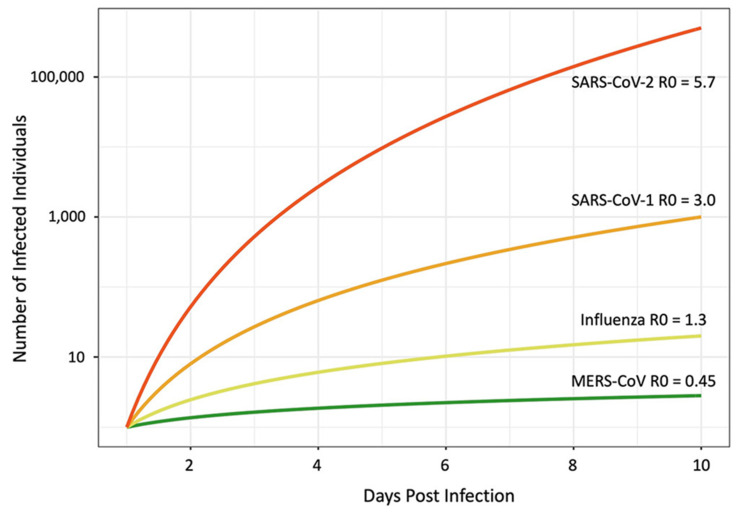
Exponential spread of recently emerged viral diseases on the basis of their R0 values. Higher R0 values indicate higher transmission rates of viral infections among human populations without acquired immunity or vaccination. The influenza virus, for which the human population has developed herd immunity, still causes seasonal flu in different parts of the world. Reprinted from the reference [25].

However, examining the early stages of the COVID-19 outbreak in China, which involved modeling travel and epidemiological data, showed a higher R0 value of approximately 5.7 [100]. Further, this value may be even higher in crowded areas such as dense urban settings; therefore, SARS-CoV-2 appears to be far more transmissible than the previously reported SARS and MERS viruses. The R0 values for MERS and SARS were 0.45 [101] and 3.0 [102], respectively. On the other hand, the R0 value of the seasonal flu is about 1.3 in a population with herd immunity. Therefore, additional studies are required to evaluate the public safety implications of accurate R0 value estimations. As discussed above, higher R0 values may result in the immediate spread of infection through an exposed population, after which the exponential stage severely caused a global pandemic crisis, as shown in Figure 1.

The further mutation or generation of new variants also influences transmission competence and R0 values; it may be low or high [64]. Some infected but asymptomatic individuals transmit the SARS-CoV-2 virus before becoming symptomatic, contributing to a higher R0 [103] and acting as super-spreaders [104]. Asymptomatic spread certainly goes undetected; however, its role in influencing the R0 value and causing rapid spread and global pandemic is well evident. However, the timely introduction of social distancing, hygiene measures, and mask-wearing have proven effective strategies for reducing R0 and associated mortality rates [99]. A high rate of transmission through asymptomatic individuals must be liable to large clusters of the SARS-CoV-2 infections, and it may perhaps result in collective immunization of the population [104]. As expected, worldwide vaccination is another potential adjunct to reduce the average R0 value. The significance of mass vaccination campaigns in different scenarios has been recently reported in Italy [105,106]. It suggests that the speed of vaccination is more important if the R0 value is higher in specific settings.

### 2.8. Viral Dose

The viral dose is a significant factor in causing a successful infection, and a low viral dose may not cause an infection. The ID_50_ value is defined as the value of viral count needed to infect 50% of the given population. Some viruses, such as influenza, have low viral doses [107], whereas those with high viral doses typically cause more severe diseases [108]. Both SARS-CoV-2 and SARS exhibited near-identical half-lives for aerosol droplet transmission and different surfaces, including plastic, copper, cardboard, and stainless steel [68]. Moreover, viruses’ viability depends on the surface material and some environmental factors [68]. However, epidemiological investigations indicate that SARS-CoV-2 possesses unique characteristics during post-infection viability, mainly with high viral loads in the upper respiratory tract [109]. Importantly, these differences enable the hidden transmission of COVID-19 during the asymptomatic phase [110]. COVID-19 infection is also possible via fomite transmission if the viral load is sufficient to cause disease since viruses remain viable in aerosol droplets [111]. The accurate estimation of the expelled SARS-CoV-2 viruses during coughing is a great challenge to the researcher community.

On the other hand, MERS and SARS virus shedding begins from symptoms, and infectivity remains till the second week from infection [112,113]. Thus, both MERS and SARS virus infections were easier for contact tracing and containment than SARS-CoV-2 viruses. However, the respiratory tract begins viral shedding from 2 to 3 days before the indication of symptoms; thus, it is evident that a large percentage of transmission occurs before the declaration of confirmatory test results [114]. COVID-19 virus shedding during the presymptomatic period and by asymptomatic patients for about 14 days is likely conceivable [115]. Thus, such cases certainly act as a significant contributor to the silent spread and global pandemic crisis, as it undergoes undetected due to limited testing capacity or delay of issuing reports [104].

### 2.9. Case Fatality Ratio (CFR)

**Figure 2:**
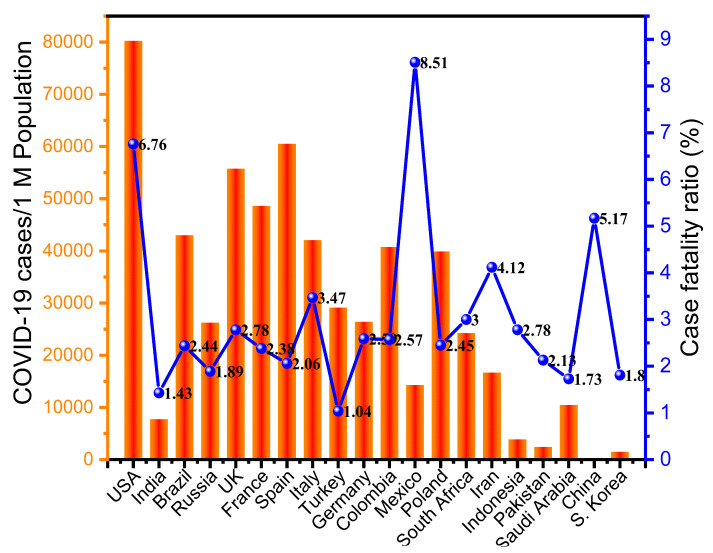
CFRs for SARS-CoV-2 in different countries as of 1 February 2021. The data were obtained from Worldometer. The graph illustrates the variations in CFR values depending on the country.

CFR of viral infections is defined as the rate of fatalities to the total count of the confirmed cases, which can be used to assess the severity [116]. The clinical complications range from asymptomatic to mild pneumonia-like symptoms or influenza viral infection-like symptoms and severe disease associated with lung tissue damage [117], multiorgan failure, and death [118]. COVID-19 survivors are also prone to a higher risk of dementia; it was more evident in females (≥60 years old) [119]. Worldometer data analysis on the first week of February 2021 revealed that the CFR for the SARS-CoV-2 virus varies among countries, as shown in Figure 2. High death rates in some countries were likely due to inadequate healthcare systems, lack of funds to handle the outbreak effectively and enough infrastructure facilities to treat patients with severe symptoms.

Furthermore, delayed or incomplete testing can result in high CFR values, or inaccurate estimates [120], since the count of the COVID-19-infected patients seems substantially higher than the number of confirmed cases after testing. The WHO’s estimates on the ratio of deaths per total confirmed cases vary from 1 to 9%, while the world average CFR value for COVID-19 is about 3.4%, which is comparatively better than SARS (11%) and MERS (34%) (Table 1). Several RNA viruses have been begun from either a bat reservoir (e.g., coronaviruses, zika virus and Ebola virus) or a bird reservoir (e.g., influenza virus), except for HIV, which evolved and originated from a primate reservoir [121] (Table 1).

Significant differences were observed in the CFR values published by different countries (Figure 2). In the initial period of the SARS-CoV-2 pandemic, most European countries exhibited considerably higher CFRs values, approximately 12.5% in the United Kingdom, 10.19% in Spain, 12.79% in Italy, 14.75% in France, 11.9% in Belgium, 10.8% in the Netherlands and 8.78% in Sweden, according to the WHO’s report (Figure 2). Among these European nations, only Germany maintained a much lower CFR value of about 2.29%. The high numbers were due to aged populations, compromised immunity, and chronic comorbidities such as diabetes, high blood pressure, and other metabolic diseases [132]. The CFR is also strongly correlated with cardiovascular diseases and the age of the infected individuals. Old age groups were more prone to develop severe complications and sometimes death after COVID-19 infection. They have weaker immune systems and often present with other conditions, such as metabolic disorders, hypertension, diabetes, and cancer [133].

Furthermore, the CFR values of a given country vary over time. Therefore, it would be challenging to make firm conclusions regarding the mortality rate [134,135] and morbidity of COVID-19. Further research might provide accurate insights into other factors contributing to high CFR values [136]. However, accessibility to well-equipped healthcare systems plays a vital role in achieving low CFR values. Notably, countries lacking experienced teams and an established healthcare infrastructure will predictably have a high CFR. Thus, as the SARS-CoV-2 global pandemic continues to spread, other countries/communities must quickly establish critical life-saving healthcare systems.

## 3. Risk Mitigation Strategies

This report briefly discusses mitigation strategies and containment measures applicable for the containment of the COVID-19 pandemic; it is a prevalent challenge to healthcare systems worldwide. The rapid development of the COVID-19 pandemic has proven that biosafety policies are a critical part of human society and economic security. At present, a constant increase in the count of the SARS-CoV-2 cases and resurgence risk is clearly evident in some counties. It is critical to protect susceptible populations by eliminating the transmission risks and avoiding superspread events. Given the current pandemic crisis, we all need to comply with the rules and regulations at the public, community, or personal levels since collective effort is a key to mitigating global COVID-19 risk. Some hazardous waste management principles are also applicable to the design framework of risk mitigation for the effective containment of SARS-CoV-2 [137]. The restoration of collaborative spirit is also essential with various allied efforts, including the safety of healthcare staff, public safety, food security, conducting surveillance, rapid detection, self-isolation, contact tracing, and medical treatments. The perspective presented here is to align with the five-stage top-down hierarchy of controls designed by the Center for Disease Control and Prevention (CDC) that implies, in descending order of practicality: elimination (isolation/quarantine), temporary options (remote work, distance learning), use of engineering controls (protect people from the exposure), implementation of administrative controls (change the behavior of people) and safeguarding with PPE [138,139]. Essentially, adopting the basis of the hierarchy of controls from an occupational safety standpoint can provide a better prospect of understanding the benefits of hazard control practices to contain the further spread of COVID-19 [139].

### 3.1. Administrative Control Measures

This account further describes the scope of administrative controls appropriate in managing disease outbreaks and public safety measures. First, administrative controls should be established as the best practices to administer any public health emergency. The lessons from the previous SARS and MERS events provided sufficient data for designing policies for administrative controls with an appropriate model of epidemiologic observations [140]. Such provisions with specific requirements for the given context have to be adopted to shun the dynamic risk of the COVID-19 pandemic. Second, standard hazard waste disposal procedures and safeguarding face masks, personal protective equipment (PPE) kits, and dressing materials protect healthcare workers should be implemented [141]. Therefore, the safe disposal of PPE kits (including gloves) used to protect the first responders, healthcare workers, and healthy patients potentially reduces the likelihood of disease dissemination in existing healthcare facilities (nursing rooms, receptionist counters, hospital departments, and other settings). The third and most crucial administrative control measure is the selection of effective disinfectant agents for COVID-19. Given that the enveloped virus SARS-CoV-2 has a phospholipid bilayer, it is susceptible to ordinary soaps, including detergents, bleaching agents, quaternary ammonium compounds, and 70% alcohol-based hand sanitizers [142,143]. These disinfectants are highly effective in dissolving the lipid layer or denaturing the SARS-CoV-2 proteins. As shown in the graphical abstract, disinfection methods are also vital to eliminate the SARS-CoV-2 viruses. Therefore, soaps, disinfectants, and hand sanitizers significantly reduce the likelihood of infection and the occurrence of infectious doses by killing the SARS-CoV-2 viruses present on surfaces.

Administrative controls also involve changing specific behaviors through policy reforms or implementing a framework to reduce the public health risk [144]. Such control measures are frequently revised to direct social distancing levels, minimize human-to-human interaction, or control human density in given spaces; such directions reduce exposure to COVID-19-infected individuals. Administrative means to consider policy reforms to restrict indoor activities, such as religious gatherings, theaters, sports stadiums, schools, and organizational-level orders promoting remote work, in-person work scheduling, and distance learning initiatives could minimize high density-indorsed risk [145]. This strategy exemplifies the major weakness of dependency on administrative authorities (the challenge here is to remain dependent on organizational management and wait for mitigation of transmission risk). The lessons learned from several contexts, patient safety concerns, risk management scenarios, and policy reforms that rely on perfect adherence, have limitations and is prone to fail. Therefore, compliance is of utmost significance for administrative controls to be effective and successful; even high-principled people sometimes make mistakes in terms of adherence to the rules. Prompt recruitment of trained healthcare professionals has also been suggested to enhance the performance of the existing healthcare system and scale-up public healthcare facilities [146,147].

### 3.2. Engineering Control Measures

Risk mitigation also depends on numerous safety measures that promote best practices for handling the SARS-CoV-2 crisis. Engineering controls refer to improving physical barriers among risk sources and health workers [146]; it is the most preferred measure to reduce transmission risk. Engineering control measures may provide solutions to ensure the physical separation of operations to treat infected persons through mechanical and physical means [63]. The best example of practical engineering controls is ventilation with a physical barrier that enables negative pressure isolation and quarantine rooms [148]. Typically, most hospital rooms are not equipped with filters such as high-efficiency particulate air (HEPA). Such air filters are applicable to retain small air particles (from 0.2 to 0.3 μm) with excellent efficiency, about 99.97% [149]. Portable HEPA filters in indoor settings can be a potential adjunct in controlling the transmission of the SARS-CoV-2 viruses. However, ventilation in conventional rooms (i.e., opening windows) has also been reported to be an effective means to reduce airborne viral loads [150]. A study conducted during the SARS epidemic revealed a significant relationship between higher ventilation in discrete isolation rooms with multiple beds and lower infection rates among healthcare workers. Increased ventilation can be an essential strategy to reduce droplet transmission, airborne aerosol transmission, and the spread of influenza in academic institutions and indoor settings [151,152]. Therefore, improving ventilation systems could be a valuable option for indoor environments. Otherwise, enhancing natural ventilation by simply opening windows also helps to increase airflow, thus decreasing the risk of infections [153]. Ensuring indoor air safety by installing ventilation and filtration systems with germicidal ultraviolet (UV) light is yet to be established under engineering controls to safeguard the indoor environments from transmission risk of COVID-19 [154,155]. The CDC further recommends additional engineering control measures, including physical barriers, partitions, UV radiation, and the use of virus-proof fabrics to avoid contact between healthy healthcare workers and infected persons. The implementation of well-designed engineering control measures was revealed to be highly effective in reducing the risk of infectious diseases to healthy individuals, despite the differences in their behaviors, and to considerably heighten the safety of individuals who complied with administrative control measures or use PPE kits [156].

### 3.3. Personal Protective Equipment (PPE)

Personal protective equipment (PPE) is essential to safeguard public healthcare workers from infectious diseases such as COVID-19. PPE is the best defensive strategy applicable in all operations; it can be used as a primary physical barrier between healthcare workers and infected patients [138]. The use of PPE kits should be prioritized, particularly when visiting isolation wards and interacting with infectious patients. The CDC has provided strict guidelines and suggested taking advantage of PPE kits; it is mandatory while contacting suspected individuals and treating patients with severe symptoms. PPE kits include disposable dresses, fit-tested N95 masks or electronic respirators, eye protectors or transparent face shields, and disposable gloves [157].

**Figure 3:**
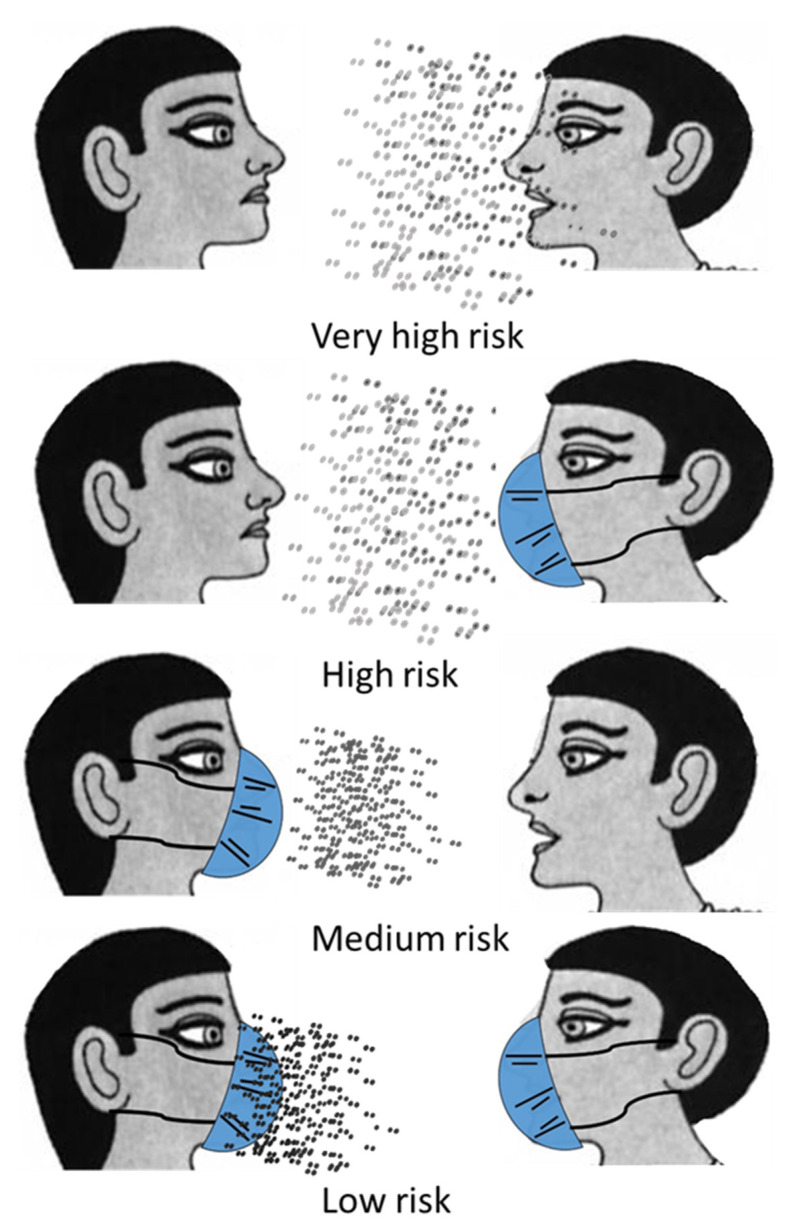
Virus transmission risk depends on the use of fit-tested N-95 masks by both infected and healthy individuals.

Furthermore, fit-tested N95 respirator masks do not filter out 100% airborne aerosol virus particles, as they are designed to act more as spray or splash barriers (Figure 3). Therefore, fit-tested N95 respirator masks effectively protect healthy individuals from infected ones, reducing transmission risk through the physical barrier to cough droplets [158]. The CDC website provides detailed guidelines on the use of fit-tested N95 respirators and masks. Respiratory protective equipment (RPE) needs to be designed to improve healthcare professionals’ protection against the inhalation of aerosol particles [159].

We further suggest considering advances in designing and manufacturing antimicrobial and antiviral functionalities on the fabric used to prepare PPE kits. It would protect the healthcare workers against viruses and bacteria and provide extra safety. Though developed countries ensured a steady supply of PPE kits, including personal protective clothing (PPC) [139], those developing countries are yet to fulfill their supply chain [160]. There is a need for further developments to ensure the supply chain [161] and safer disposal of single-use fabrics used in protective clothing to reduce negative impacts on the environment [162]. Future research developments should consider ways to increase safety, efficiency, and accessibility of PPC globally with reduced impact on the environment.

### 3.4. Herd Immunity via Vaccination

“Herd immunity”, defined as “population immunity”, is the indirect protection of people in a population or the prevention of infectious diseases that ensue when most community members are immune via infection or vaccination. Most authorities impelled to accomplish “herd immunity” via vaccination rather than letting the disease spread to a given section of the population. Since it can result in excessive infections and deaths, there is the risk of a public health crisis. Besides this, vaccines develop short-term or long-term immune responses by instant-forming antibodies against future disease events. An active infection can occur in the future or cause disease, but importantly, the vaccination helps to recover without causing severe illness [163,164].

Vaccines have effectively controlled historical contagious diseases such as polio, smallpox, rubella, diphtheria, and many others [165]. COVID-19 vaccine distribution initiatives have begun, and vaccination rates have increased around the world [166]. However, people have reasonably started to wonder when this pandemic will end. Most of the time, the answers from experts and even authorities seem to be full of uncertainties. There is an intense expectation that, at one point, enough people will eventually gain immunity against SARS-CoV-2 that it will break the transmission chain (i.e., we will reach the “herd immunity threshold”), but, so far, it appears doubtful [167]. Nevertheless, herd immunity of COVID-19 through vaccination might be difficult or impossible for several reasons, including vaccination hesitancy, protection-related questions, and uneven vaccine rollout [168].

First, some individuals object to receiving the COVID-19 vaccine because of religious reasons, fears about the health risks (such as allergic reactions), hesitancy over vaccine acceptance, and skepticism over benefits [169]. Thus, if the proportion of vaccinated people in a community is lower than the “herd immunity threshold,” the transmissible disease will continue to spread [167]. Appropriately, a large proportion of the global population needs to be vaccinated against COVID-19 to instigate herd immunity. Second, protection-related questions have raised the critical issue of confusion about how long the COVID-19 vaccines will protect against COVID-19 [170]. Recent research also suggests that current COVID-19 vaccines may have minor efficacy against some of the newly emerging variants of the COVID-19 virus, which can be resistant or non-responsive [171]. Third, the distribution of COVID-19 vaccines and vaccination rates varies significantly among countries, states, and local vaccination centers [172]. Even if a particular community achieves a high COVID-19 vaccination rate, outbreaks will repeatedly occur in the population mix if nearby areas do not. Overall, this is an important research area, and vaccine distribution will likely differ among communities; therefore, integrated vaccination and physical distancing interventions need to be redirected [172]. The key factors that make achieving “herd immunity” challenging include vaccine type/effectiveness/distribution sustainability, prioritized populations for vaccination, and several other factors [173,174]. Further investigation is necessary to examine the safety and efficacy of the SARS-CoV-2 vaccines’ potential to reduce the transmission and spread of the virus [175,176].

The rapid progress of vaccination programs against COVID-19 signifies colossal contemporary accomplishment and offers new hope of culminating the global pandemic crisis [177]. Presently, several countries are making progress toward “herd immunity” by adopting the ethical route of massive-scale vaccinations. As the vaccination drives take off at incredible speeds, the number of fully vaccinated adults continues to rise, but the answer to the critical question remains unclear—how long immunity will last after infection or vaccination [178]. Therefore, among several challenging aspects, it is not clear if or when a particular country will achieve the goals of herd immunity. Most of the approved COVID-19 vaccines can be highly effective at protecting the population against serious health complications, thus reducing the number of patients needing to be hospitalized and achieving lower mortality rates [179,180]. Although it would be impossible to contain the transmission of the COVID-19 virus completely, successful vaccination programs will allow humans to live more comfortable life with the COVID-19 pandemic.

## 4. Experimental Section

We here examine the observational, prospective, epidemiological studies in scientific literature. The primary objective of this report is to raise awareness on public health risks and suggest appropriate mitigation strategies. Thus, the SARS-CoV-2 pandemic is examined using an investigator’s perspective of risk assessment and mitigation based on recent developments in the literature and is related to the public health approach. This report explores the reason behind why COVID-19 accelerated progressively in the direction of a global pandemic, which affected our human lives and several sectors of the economy to a degree not known in the recent past.

A formal method is used to evaluate multiple aspects of risk assessments for the SARS-CoV-2 pandemic, e.g., implications for humans and the environment. Risk assessments are commonly performed in research environments, particularly for drug development, and the safety of hazard materials is considered in this report. The basis of risk assessment involves several well-defined means, including the biological characteristics of the pathogen, susceptibility of the human host, and the context of public health or impacts on the environment. However, it is critical to relate public health aspects while performing an assessment of severe public health concerns of a newly emerged infectious disease. This report is to aid further progress of public awareness, policy reforms, and prompt healthcare decisions in the realm of public health and risk mitigation strategies, including administrative control measures, engineering control measures, and personal protective equipment.

## 5. Conclusions

Influenza and other coronavirus outbreaks, including SARS and MERS, have few features in common: (a) human interaction with wildlife animals, interspecies transmission, mutations, and human-to-human transmission, and (b) global pandemic reach. Unlike SARS-CoV-2, SARS and MERS were timely controlled and contained before taking the shape of public health and global pandemic crisis. COVID-19 is a highly transmissible disease; viral shedding manifests prior to the onset of noticeable symptoms. Several patients remain asymptomatic and act as super-spreaders, continuing to infect other healthy individuals. Thus, SARS-CoV-2 undoubtedly spreads silently, threatening public safety globally, particularly in high mobility and population density settings.

The perspective on risk assessment for SARS-CoV-2-like infections is indispensable in designing policies for administrative and engineering controls and PPE kits. Risk awareness could be the best approach to contain the transmission rate and relax the SARS-CoV-2 pandemic crisis. This article discusses risk assessment perspectives to increase the public health awareness of SARS-CoV-2, improve decision making, reduce the negative impact of excess communication and provide directions to deal with current challenges. There is a need to convey potential risk assessment perspectives relevant to SARS-CoV-2, with profound reach and clarity, as well as introducing new behaviors, strengthening social compliance, lowering exposure risk, establishing possible interventions, and simultaneously reducing misconceptions. Further, perspectives on risk assessment are essential to implement the potential interventions and effective management of pandemics, together with enabling an inclusive response to contain the resurgence.

Presently, several countries are working proactively to save lives, establish medical infrastructure, mobilize vaccines to citizens and make serious efforts to limit losses to several sectors and economies, ultimately preventing expected regional or global economic recession. The context- and country-specific lessons learned so far from the SARS-CoV-2 pandemic need to be applied to devise preventive actions against the resurgence of SARS-CoV-2 and safeguard future pandemic circumstances. Therefore, there is a need to consider the conceptual design of the framework, development of medical infrastructures, and promotion of non-pharmaceutical interventions to fast-track the performance of risk mitigation strategies and ensure the containment of future pandemics.

## Figures and Tables

**Table 1:** Previous and recent pandemic viral diseases worldwide and their fatality ratios.

Year	Contagion	Disease	Worldwide Cases	Worldwide Deaths	Fatality Ratio	References
1918	Influenza A (H1N1)	Influenza	500 million	>17.4 million	>2.54%	[122]
1957–1959	Influenza A (H2N2)	Influenza	unidentified	1.1 million	<0.11%	[123]
1968	Influenza A (H3N2)	Influenza	unidentified	1.0 million	<0.52%	[124]
1981	HIV	HIV/AIDS	75 million	32 million	99.98%	[125]
2002	SARS	SARS	8422	916	11.4%	[126]
2009	Influenza A (H1N1)	Influenza	12,700	4700	0.1–5%	[127]
2012	MERS	MERS	2494	11,325	34%	[128]
2014–2016	Ebola virus	Ebola	28,652	13,562	40%	[129]
2016	Zika virus	Zika	41,300	---	8.3%	[130]
2019	SARS-CoV-2	COVID-19	101,561,219	2,196,944	2.1	[131]

## Data Availability

Not applicable.
